# Supplementary material for: Yiai Fuzheng decoction inhibits triple-negative breast cancer by remodeling the immune microenvironment
Source: Front Immunol. 2025 Sep 30;16:1615631. doi: 10.3389/fimmu.2025.1615631 (PMC12518410; doi:10.3389/fimmu.2025.1615631)
Supplement: Supplementary file 2 [file DataSheet1.zip › Supplementary File 2/Treatment/C_vs_H/07_Pathway_Analysis/Pathway_Analysis/Sig_Pathways_Name/Alanine, aspartate and glutamate metabolism.pdf]

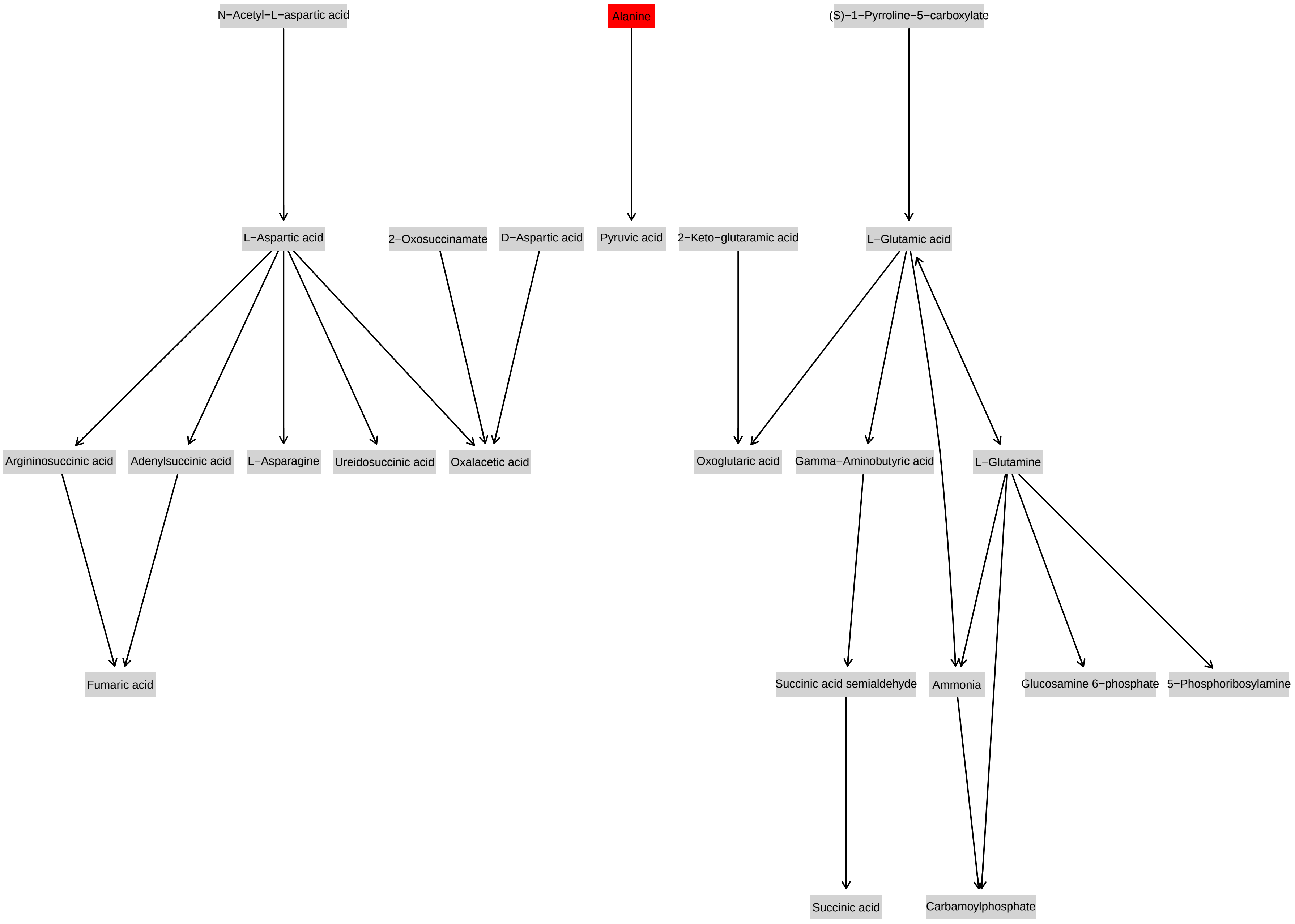
N−Acetyl−L−aspartic acid
Alanine
(S)−1−Pyrroline−5−carboxylate
L−Aspartic acid
2−Oxosuccinamate
D−Aspartic acid
Pyruvic acid
2−Keto−glutaramic acid
L−Glutamic acid
Argininosuccinic acid
Adenylsuccinic acid
L−Asparagine
Ureidosuccinic acid
Oxalacetic acid
Oxoglutaric acid
Gamma−Aminobutyric acid
L−Glutamine
Fumaric acid
Succinic acid semialdehyde
Ammonia
Glucosamine 6−phosphate
5−Phosphoribosylamine
Succinic acid
Carbamoylphosphate
